# RAD52 S346X variant reduces breast cancer risk in *BRCA2* mutation carriers

**DOI:** 10.1002/1878-0261.12679

**Published:** 2020-04-21

**Authors:** Kajal Biswas, Shyam K. Sharan

**Affiliations:** ^1^ Mouse Cancer Genetics Program Center for Cancer Research National Cancer Institute National Institutes of Health Frederick MD USA

## Abstract

Adamson *et al*. report that *BRCA2* mutation carriers inheriting *RAD52 S346X* variant have reduced breast cancer risk. The RAD52 S346X variant lacks the nuclear localization sequence, which mislocalizes the protein to the cytoplasm and renders it nonfunctional. Combined loss of BRCA2‐mediated DNA repair by homologous recombination and RAD52‐mediated single‐strand annealing may result in cell death and reduce breast cancer risk.

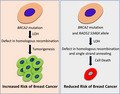

Comment on: https://doi.org/10.1002/1878-0261.12665

**Abbreviations**
CIMBA: Consortium of Investigators of Modifiers of *BRCA1* and *BRCA2*
DSB: double‐strand break
HR: homologous recombination
SSA: single‐strand annealing

BRCA1 and BRCA2 are the two well‐known tumor suppressors, and their mutations are associated with increased risk of breast and ovarian cancers (Samadder *et al.*, 2019). It is well known that individual risks of *BRCA1/2* mutation carriers can vary due to a number of factors, and additional genetic changes or genetic modifiers that can modify tumor predisposition (Friebel *et al.*, 2014). Genomewide association studies have identified a number of loci that alter the breast cancer risk in *BRCA1/2* mutation carriers (Milne and Antoniou, 2011). A noncoding polymorphism at 5′UTR in *RAD51* has been shown by multiple independent studies to increase breast cancer risk in *BRCA2* carriers (Antoniou *et al.*, 2007). RAD51 is the key protein that interacts with both BRCA1 and BRCA2 and is required for the repair of double‐strand breaks (DSBs) by homologous recombination (HR) (Pellegrini and Venkitaraman, 2004).

In a recent study, a truncating variant of RAD52 has been found to significantly reduce the breast cancer risk in *BRCA2* mutation carriers, which supports a role for RAD52 as a genetic modifier of cancer predisposition associated with BRCA2 loss (Adamson *et al.*, 2020). Originally identified in *Saccharomyces cerevisiae*, Rad52 was found to be an essential gene that is required for HR by facilitating the recruitment of Rad51 onto replication protein A‐coated single‐stranded DNA (Symington, 2002). In higher organisms, BRCA2 has a more important role in RAD51 recruitment and RAD52 is required for an alternate DNA repair pathway known as single‐strand annealing (SSA) (Jalan *et al.*, 2019). The importance of RAD52‐dependent SSA pathway was highlighted by work from the Powell laboratory, when they showed that RAD52 loss results in synthetic lethality of BRCA2‐deficient cells (Feng *et al.*, 2011). BRCA1‐deficient cells also exhibit synthetic lethality in response to RAD52 inactivation (Lok *et al.*, 2013). The mechanism of synthetic lethality was presumed to be the loss of multiple pathways of DSB repair, including the HR and SSA pathways. Recent studies have revealed the involvement of RAD52 in additional cellular processes, which may also contribute to the synthetic lethality (Jalan *et al.*, 2019). The new functions of RAD52 include its role in break‐induced replicative stress response, where it is required for the restart of collapsed replication forks, as well as in a subset of microhomology‐mediated break‐induced replication known as MiDAS (mitotic DNA synthesis) (Bhowmick *et al.*, 2016; Sotiriou *et al.*, 2016).

In the study by Adamson *et al.* (2020), the presence of the S346X truncating variant of RAD52 was found to be associated with a significant reduction in the risk of breast cancer in *BRCA2* mutation carriers. The authors identified S346X as a common variant of *RAD52*, with a minor allele frequency of 0.017. This allowed them to investigate its impact on breast and ovarian cancer risk in individuals carrying pathogenic *BRCA1* and *BRCA2* mutation. Out of the 10 979 *BRCA2* mutation carriers they identified, 5605 were diagnosed with breast cancer and 2369 with ovarian cancer. In these carriers, the presence of *RAD52 S346X* allele was significantly associated with reduced risk of breast cancer: 159 *RAD52 S346X* heterozygotes did not have breast cancer compared to 118 *RAD52 S346X* heterozygotes who had breast cancer. One *BRCA2* mutation carrier who was homozygous for the minor *RAD52* allele had breast cancer, and three homozygotes did not have breast cancer. Statistical analysis resulted in a hazard ratio of 0.69 (95% CI = 0.56–0.86, *P* = 0.0008). In simple terms, hazard ratio represents the ratio of the incidence of breast cancer in carriers of *RAD52 S346X* minor allele and those without the minor allele. The risk reduction was also examined in 15 679 *BRCA1* mutation carriers, but the impact of *RAD52 S346X* allele was found to be less in *BRCA1* mutation carriers (hazard ratio: 0.78, 96% CI = 0.64–0.97, *P* = 0.02). Interestingly, the impact of the *RAD52 S346X* allele on ovarian cancer was similar to that observed for breast cancer, but the hazard ratios were not significant because of the smaller sample size.

The RAD52 S346X variant retains all the functional domains, and the mutant protein is likely to be proficient in DNA binding and strand annealing (Adamson *et al.*, 2020). However, it lacks the last eight amino acids that encode the nuclear localization sequence. Loss of these amino acids was shown to render the protein nonfunctional because it localized predominantly in the cytoplasm instead of the nucleus (Adamson *et al.*, 2020). The authors found S346X variant to have significantly reduced SSA activity using a GFP‐based reporter in mouse ES cells. Furthermore, the authors showed that knockdown of BRCA2 increased SSA levels in cells expressing WT RAD52. By contrast, BRCA2‐deficient cells expressing the RAD52 S346X variant were found to suppress SSA. Thus, lack of functional RAD52 may reduce the mutagenic effects of SSA and contribute to tumor suppression. Alternatively, it is possible that cells undergoing loss of heterozygosity in *BRCA2* mutation carriers undergo apoptosis in the presence of RAD52 S346X variant, due to the persistence of unrepaired DSBs that may suppress tumorigenesis and reduce cancer risk. The later possibility is supported by the RAD52 loss‐mediated synthetic lethality of BRCA2‐deficient cells (Feng *et al.*, 2011).

The synthetic lethality caused by RAD52 inactivation in BRCA1/2‐deficient cells has made RAD52 a viable therapeutic target (Toma *et al.*, 2019). The fact that RAD52 is dispensable for normal growth and development of mice has made it even more attractive target (Rijkers *et al.*, 1998). Use of RAD52 inhibitors for targeted treatment of BRCA‐deficient tumors is being explored. A number of small‐molecule inhibitors of RAD52 have been identified (Toma *et al.*, 2019). Several of these have been shown to be effective in inhibiting the growth of BRCA‐deficient cells. More recently, RAD52 inhibitors were shown to be effective in targeting BRCA1‐deficient tumor growth in mouse xenograft models (Sullivan‐Reed *et al.*, 2018). Furthermore, these inhibitors had a synergistic effect when combined with PARP inhibitors (Sullivan‐Reed *et al.*, 2018).

The present finding that RAD52 S346X reduces cancer risk in *BRCA* mutation carriers suggests that RAD52 inhibitors may also be used to reduce breast cancer risk in *BRCA1/2* mutation carrier. The impact of RAD52 inhibition on tumor suppression needs to be further validated before inhibitors can be tested for cancer prevention in *BRCA1/2* mutation carriers. The toxicity and impact of long‐term use of such inhibitors will have to be carefully tested before any prevention studies can be initiated.

The risk assessment of *BRCA1/2* mutation carriers inheriting other RAD52 variants that disrupt the protein function may also identify other alleles that are associated with reduced cancer risk. Similarly, search for other genetic modifiers may reveal other avenues for cancer treatment and prevention. Such challenging projects are largely dependent on the global collaborative efforts, such as the Consortium of Investigators of Modifiers of *BRCA1* and *BRCA2* (CIMBA, http://cimba.ccge.medschl.cam.ac.uk/), that are focused on identification of new BRCA1/2 mutation risk modifiers.

## Conflict of interest

The authors declare no conflict of interest.
